# Petroleum contaminated water and health symptoms: a cross-sectional pilot study in a rural Nigerian community

**DOI:** 10.1186/s12940-015-0073-0

**Published:** 2015-11-06

**Authors:** Kalé Zainab Kponee, Andrea Chiger, Iyenemi Ibimina Kakulu, Donna Vorhees, Wendy Heiger-Bernays

**Affiliations:** Department of Epidemiology, Harvard University School of Public Health, Boston, MA 02115 USA; Department of Environmental Health, Harvard University School of Public Health, Boston, MA 02115 USA; Department of Environmental Health, Boston University School of Public Health, 715 Albany St. T4W, Boston, MA 02118 USA; Department of Estate Management, Rivers State University of Science and Technology, Port Harcourt, Rivers State Nigeria

**Keywords:** Petroleum hydrocarbons, Drinking water, Contamination, Public health, Refined oil, Adverse health effects

## Abstract

**Background:**

The oil-rich Niger Delta suffers from extensive petroleum contamination. A pilot study was conducted in the region of Ogoniland where one community, Ogale, has drinking water wells highly contaminated with a refined oil product. In a 2011 study, the United Nations Environment Programme (UNEP) sampled Ogale drinking water wells and detected numerous petroleum hydrocarbons, including benzene at concentrations as much as 1800 times higher than the USEPA drinking water standard. UNEP recommended immediate provision of clean drinking water, medical surveillance, and a prospective cohort study. Although the Nigerian government has provided emergency drinking water, other UNEP recommendations have not been implemented. We aimed to (i) follow up on UNEP recommendations by investigating health symptoms associated with exposure to contaminated water; and (ii) assess the adequacy and utilization of the government-supplied emergency drinking water.

**Methods:**

We recruited 200 participants from Ogale and a reference community, Eteo, and administered questionnaires to investigate water use, perceived water safety, and self-reported health symptoms.

**Results:**

Our multivariate regression analyses show statistically significant associations between exposure to Ogale drinking water and self-reported health symptoms consistent with petroleum exposure. Participants in Ogale more frequently reported health symptoms related to neurological effects (OR = 2.8), hematological effects (OR = 3.3), and irritation (OR = 2.7).

**Conclusions:**

Our results are the first from a community relying on drinking water with such extremely high concentrations of benzene and other hydrocarbons. The ongoing exposure and these pilot study results highlight the need for more refined investigation as recommended by UNEP.

## Background

While dramatic events such as the 1989 Exxon Valdez and 2010 Deepwater Horizon oil spills have received substantial remediation responses, the long-term and vast contamination of the Niger Delta region remains largely un-remediated [1]. Extraction, processing, and transport of crude oil dating back to the 1950s have had a devastating impact on Ogoniland, a territory in the Southern region of Nigeria. The United Nations Development Programme estimates that 6178 oil spills occurred in Ogoniland between 1976 and 2011, resulting in discharges of approximately three million barrels of oil [2]. Commissioned by the Federal Government of Nigeria, the United Nations Environmental Programme (UNEP) conducted an environmental assessment of Ogoniland and determined that the widespread oil contamination presents serious threats to human health [3]. Residents are exposed to petroleum hydrocarbons that are released to the environment by burning, spilling, and leaking. Exposure can occur via inhalation of hydrocarbons in ambient air and via consumption of and dermal contact with hydrocarbons in water, soil and sediment [1, 3].

This study focuses on the community of Ogale, located in the Eleme local government area of Ogoniland, where UNEP discovered substantial leakage from an abandoned section of a pipeline carrying refined oil. UNEP testing revealed approximately three inches of refined oil floating on the groundwater that supplies the community’s drinking water [3]. UNEP detected numerous petroleum hydrocarbons in water from individual borehole drinking water wells, notably benzene at concentrations as high as 9280 micrograms per liter, which is approximately 1800 times higher than the United States Environmental Protection Agency (US EPA) drinking water standard and over 900 times higher than the World Health Organization (WHO) drinking water guideline [3, 4].

Petroleum products are a complex mixture of hydrocarbons, consisting of both aromatic and long- and short-chain aliphatic hydrocarbons. Components of crude and refined petroleum, namely volatile organic compounds (VOCs), such as benzene, toluene and xylenes, and polycyclic aromatic hydrocarbons (PAHs), have independently been associated with adverse human health effects. Acute exposures to high concentrations of VOCs cause central nervous system toxicity, resulting in symptoms such as headaches, fatigue and dizziness [5, 6]. Chronic exposure to VOCs can impair the immune system via oxidative stress and decreases in white blood cell count [7, 8]. Benzene in particular is strongly associated with disorders of the hematopoietic system such as aplastic anemia [9, 10, 11]. Benzene is also classified as a known human carcinogen based on occupational studies in humans [4]. Polycyclic aromatic hydrocarbons cause symptoms such as nausea, vomiting and skin and eye irritation following acute, high-level exposures [12, 13]. Exposures to PAHs during pregnancy have been linked to decreased birth weight and impaired development in offspring [14]. Chronic occupational exposures are associated with dose-dependent increased risks of certain types of cancers, including lung, skin and bladder cancer [15]. Naphthalene, a low molecular weight PAH that was detected in Ogale water samples, can adversely affect the hematopoietic system, damaging and killing red blood cells, causing symptoms such as shortness of breath and fatigue [12, 16]. Alkylated PAHs comprise the majority of PAHs detected in petroleum products and are particularly persistent. Although the health effects of alkylated PAHs have not been well studied, limited evidence suggests that they may be more toxic and carcinogenic than their parent PAH compounds [17].

Although UNEP did not complete a detailed chemical characterization of the refined oil in Ogale wells, studies on petroleum exposures may provide some indication of adverse health effects that could occur in the community. Prior research has primarily focused on high-dose, short-term occupational exposures to crude oil, in particular those occurring during remediation of oil spills. Workers exposed to petroleum hydrocarbons have reported adverse health symptoms such as headaches, eye and skin irritation and respiratory difficulties [18, 19]. A recent cross-sectional study found that blood samples of oil spill workers showed alterations consistent with impairment of the hepatic and hematopoietic systems [20]. Research on the *Prestige* oil spill has provided preliminary evidence of exposure-dependent DNA damage in cleanup volunteers [21]. The ongoing NIEHS Gulf Long Term Follow Up (GuLF) Study on Deepwater Horizon spill workers will be the first to investigate long-term physical health effects using a prospective cohort design [22, 23].

Few studies have examined adverse effects associated with chronic exposure to elevated concentrations of refined oil products in the general population. Increases in depression and stress, stemming from perceived health risks and financial concerns, have been observed in communities subjected to chronic oil spill exposures [24]. One study found increases in cancer incidence and mortality in communities near the Amazon basin oil fields in Ecuador [25]. A thorough search of the scientific literature revealed only one health study conducted in the Niger Delta region, which reported higher rates of respiratory and skin disorders in Eleme compared to a less-industrialized Nigerian community [26]. However, this study does not include a description of the sampling design and locations, among other study weaknesses, and is therefore not suitable for reaching conclusions regarding any association between petroleum exposure and adverse health outcomes. Further research on chronic, high-magnitude exposures in individuals living in proximity to oil sites is needed to improve understanding of how oil spills might affect human health.

UNEP made several recommendations in the Ogoniland environmental assessment, including provision of alternative drinking water supplies to Ogale, remediation of soil and groundwater contamination in the area, medical surveillance, and monitoring for potential adverse health effects through the implementation of a prospective cohort epidemiological study [3]. The objectives of this pilot study are to (i) follow up on UNEP recommendations by investigating health symptoms associated with exposure to contaminated water; and (ii) assess the adequacy and utilization of an emergency supply of potable water provided by the government. We compared the prevalence of self-reported health symptoms in Ogale and in a reference community, Eteo. The results of this pilot study will be helpful for designing the prospective cohort study in Ogale recommended by UNEP.

## Methods

### Study population

The community of Ogale was selected for study based on the UNEP environmental assessment, which identified Ogale as having the most serious groundwater contamination observed in Ogoniland [3]. The community of Eteo was chosen to serve as a reference group because it is near Ogale (approximately 10 miles away), it is part of the same local government area of Eleme, and people living in Eteo and Ogale are comparable with respect to race, language, culture, and behavioral practices. The UNEP environmental assessment did not report any petroleum contamination in Eteo.

A total of 200 adults over the age of 18 were enrolled in this pilot study (100 participants from each community). We employed a stratified random sampling strategy through door-to-door recruitment in three areas of both Ogale and Eteo, approaching individuals in every fifth house. In both communities, we obtained a 98 % response rate. Participants met the following eligibility criteria: 1) residence in the community for a minimum of one year, and 2) no prior history of residence in any other Ogoniland community associated with high levels of petroleum contamination, as reported by UNEP [3].

This study was conducted with approval from local authorities in Ogale and Eteo and from the Institutional Review Board at Boston University Medical Center (reference number: H-32345). Informed consent was obtained from each subject.

### Outcome ascertainment

Trained interviewers administered standardized questionnaires in each respondent’s home. These questionnaires were developed for this pilot study and include primarily closed-ended questions regarding demographics, smoking habits, water supply, water safety, current health symptoms and medical history. Participants were asked to report their primary water source and duration of its use for specific household activities: bathing, cooking, washing, drinking, brushing teeth, cleaning the house, and washing clothes, dishes and food. We collected information on primary source water characteristics such as odor and perceived safety. We asked individuals in Ogale who reported receiving emergency government-supplied water about the duration, frequency and sufficiency of water delivery. Participants who reported currently experiencing health issues were asked to list their symptoms; interviewers were careful not to lead participants in the open-ended responses.

### Statistical analysis

Descriptive statistics were used to compare demographic information of participants in Ogale and Eteo, and chi-square analyses were used to compare frequencies of self-reported perceptions of water odor and safety between communities. The relationship between exposure to contaminated water and self-reported adverse health outcomes was studied in distinct multivariate logistic regression models, which were used to obtain odds ratios and 95 % confidence intervals (CI) for self-reported symptoms in both communities. Sex, age, occupation, smoking status, and education were fitted in one multivariate logistic regression model. Due to our small sample size, only age, sex, and smoking status were included in the detailed logistic regression model to avoid over-fitting the multivariate model. These covariates were selected because of their potential to confound or modify the association of interest [27–30]. All analyses were performed using SAS statistical software version 9.3 (SAS Institute, Cary, NC).

## Results

Participants in Ogale and Eteo were comparable in demographic characteristics (Table 1). Mean ages in Ogale and Eteo were 34.3 (SD = 11.2) and 35.5 (SD = 12.7) respectively. In our sample, there were slightly higher proportions of women (53 % in Ogale, 55 % in Eteo) than men in both communities. More than one-half of participants in Ogale and Eteo were married (60 and 71 % respectively). The vast majority of study participants sampled identified as Christian (98 % in Ogale, 95 % in Eteo).

**Table 1:** Characteristics of participants in Ogale and Eteo

Characteristic	Ogale	Eteo
	(*n* = 100)	(*n* = 100)
Age, Mean (SD)	34.3 ± 11.2	35.5 ± 12.7
Age category		
18–20	3	5
21–40	80	70
41–60	11	19
> 60	6	6
Sex (%)		
Female	53	55
Male	47	45
Religion (%)		
Christian	98	95
Muslim	2	5
Marital status (%)		
Married - Monogamous	60	71
Married - Polygamous	2	0
Widowed	3	5
Single	35	24
Smoking (%)		
Never smoker	85	89
Ever smoker	15	11
Education level (%)		
No formal education	1	3
Primary school	7	18
Secondary school	53	85
Post-Secondary school	39	21
Occupation of head of household (%)		
Farmer	6	15
Educator	3	0
Artist/Musician	2	0
Tradesman	40	44
Professional	14	9
Government/Civil Service	22	10
Clergy	2	1
Student	3	8
Unemployed	4	9
Retired	4	4
Primary medical care location (%)		
Private clinic	36	14
Primary health center	8	41
General hospital	26	20
Local chemist	19	15
None	8	8
Other	3	2
Residents in household (median)	4	5
Number of children^a^ in household (median)	2	3

*Abbreviations*: *SD* Standard Deviation

^a^Defined as individuals under the age of 18

In both communities, more than three-quarters of participants reported never having smoked tobacco (85 % in Ogale, 89 % in Eteo) and of those who did, the overwhelming majority was male. On average, participants in Ogale had achieved higher levels of education than those in Eteo; nearly twice as many Ogale participants reported post-secondary education (39 % vs. 21 %). Head of household occupations were similar across both groups. The most commonly reported occupation for the head of household in both communities was a tradesman (40 % in Ogale, 44 % in Eteo). Both communities had comparable median numbers of total individuals (4 in Ogale, 5 in Eteo) and of children (2 in Ogale, 3 in Eteo) residing in the household. Participants in both communities had consistent access to medical services. The majority of participants reported visiting a health centre, general hospital or private clinic for their medical care and health services (72 and 76 % in Ogale and Eteo respectively). In addition, 19 % of participants in Ogale sought medical care from a local chemist compared to 15 % in Eteo.

The prevalence of emergency water use as a primary source for individual household activities in Ogale ranged between 14 and 16 % (Table 2). Emergency water was most commonly used for drinking, cooking, brushing teeth, and washing food. The majority of participants in Ogale reported continued use of the contaminated water: 66 % reported drinking, 81 % reported cooking, and 83 % reported using their borehole well water for bathing, washing food, washing dishes, washing clothes, and cleaning the house. Additional reported sources of drinking water in Ogale were sachet water, bottled water and mono-pump (14, 4 and 1 % respectively). Only 24 % of participants in Ogale reported receiving emergency water supplies. Although over 80 % of these individuals stated that water delivery occurs at least once per week, half of them found the volume of water delivered to be insufficient for daily needs.

**Table 2:** Primary sources of water for various household activities in Ogale (*n* = 100)^a^

	Primary water source (*n*)		
Household activity	Borehole	Sachet^b^	Emergency^c^
Cooking	81	0	16
Drinking	66	14	15
Brushing teeth	80	1	16
Bathing	83	0	15
Washing food	83	0	16
Washing dishes	83	0	15
Washing clothes	83	0	14
Cleaning house	83	0	14

^a^Water sources with *n* < 5 (dugout well, monopump, bottled water and other) were excluded

^b^Sachet water is defined as water packed in plastic bags, commonly sold in outdoor markets

^c^Emergency water is defined as water supplied by the government to Ogale participants as requested by UNEP

Eteo residents are not provided with emergency water because their water supply is not known to be contaminated. Approximately 97 % of individuals in Eteo reported using their individual household borehole drinking water wells for all specified household activities, while 2 % used surface water, and 1 % used rain water. Overall, participants in Ogale reported using their borehole well water for household activities for a median of 4 years, while participants in Eteo reported a median of 5 years. The median years of exposure to emergency drinking water and to sachet water in Ogale were 1 and 2, respectively.

Participants in Ogale were significantly more likely to perceive their primary water source as having an odor (39 % vs. 8 %) The main sources of water with a reported odor in Ogale were borehole well and emergency water (Table 3). The majority of participants in Ogale who reported a borehole well odor (69 %) stated that it smelled like petroleum fuel. Among Ogale participants reporting an emergency water odor, the most common description was chlorine (10 %). In Eteo, one participant described the borehole well water as having a fuel odor (1 %).

**Table 3:** Detailed descriptions of water odor in Ogale and Eteo

	Water location and source (*n* (%))				
	Ogale (*n* = 39)			Eteo (*n* = 8)	
Odor description	Borehole	Sachet	Emergency	Borehole	Sachet
Any odor	32 (82.1)	1 (2.6)	6 (15.4)	6 (75.0)	2 (25.0)
Fuel	27 (69.2)	1 (2.6)	1 (2.6)	1 (12.5)	0
Chlorine	0	0	4 (10.3)	0	0
Chemical	2 (5.1)	0	1 (2.6)	0	1 (12.5)
Mold	0	0	0	1 (12.5)	0
Mud	1 (2.6)	0	0	0	0
Unpleasant	0	0	0	3 (37.5)	0
Smoke	1 (2.6)	0	0	0	0
Don’t know	1 (2.6)	0	0	1 (12.5)	1 (12.5)

**Fig. 1:**
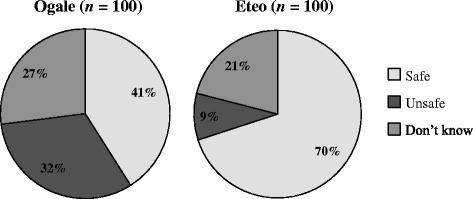
Perceptions of primary water source safety in Ogale and Eteo. Participants from both communities reported that their primary water source was safe (*light gray*), unsafe (*medium gray*), or they did not know if it was safe or not (*dark gray*)

When asked about water safety, 41 % of participants in Ogale reported perceiving their water source as “safe” compared to 70 % in Eteo (Fig. 1). In addition, almost one-third (32 %) of individuals in Ogale reported their water as “unsafe” compared to 9 % in Eteo. Differences in these proportions were found to be statistically significant at *p* < 0.05 level using chi-square analyses.

Table 4 displays differences in symptom reporting across the three different primary sources of drinking water (borehole, sachet, and emergency water) in Ogale. On average, the proportions of individuals who reported experiencing current adverse health symptoms were similar among the three different sources of primary drinking water in Ogale. Overall, chi-square tests revealed no statistically significant differences in the proportions of health symptoms reported among individuals who used borehole, sachet, or emergency water for drinking.

**Table 4:** Self-reported adverse health symptoms by primary drinking water sources in Ogale^a^

	Primary drinking water source (*n* (%))		
	Borehole	Sachet	Emergency
Health Symptom	(*n* = 66)	(*n* = 14)	(*n* = 15)
Irritation	34 (51.5)	7 (50.0)	6 (40.0)
Neurologic	27 (40.9)	5 (35.7)	8 (53.3)
Gastrointestinal	10 (15.2)	2 (14.3)	5 (33.3)
Hematologic	21 (31.8)	5 (35.7)	3 (20.0)
General pain	6 (9.1)	0 (0.0)	2 (13.3)

^a^Differences in proportions are not statistically significant at *P* < 0.05 using chi-square analyses

Chi-square analyses were used to examine differences in health symptoms among participants in Ogale who reported receiving sufficient, insufficient or no emergency water supplies (Table 5). The frequency of irritation and gastrointestinal symptoms were significantly different between the three groups. Participants in Ogale who received sufficient emergency water supplies were less likely to report having irritation (8.3 %) than those receiving insufficient or no emergency water (66.7 and 50 % respectively). Overall, participants who received sufficient emergency water supplies reported the lowest proportions of health symptoms across the three groups.

**Table 5:** Self-reported adverse health symptoms by sufficiency of emergency water supply in Ogale

	Sufficiency of water supply (*n* (%))		
	Sufficient	Insufficient	No supply
Health symptom	(*n* = 12)	(*n* = 12)	(*n* = 76)
Irritation*	1 (8.3)	8 (66.7)	38 (50.0)
Neurologic	3 (25.0)	8 (66.7)	29 (38.2)
Gastrointestinal*	1 (8.3)	6 (50.0)	10 (13.2)
Hematologic	3 (25.0)	5 (41.7)	22 (30.0)
General pain	1 (8.3)	1 (8.3)	6 (7.9)

*Differences in proportions are statistically significant at *P* < 0.05 using chi-square analyses

Table 6 displays general and specific self-reported health symptoms for all participants. After controlling for age, sex, smoking status, occupation, and education level, Ogale residents were significantly more likely to self-report any irritation (OR = 2.7; 95 % CI, 1.5–5.1), any neurological effects (OR = 2.8; 95 % CI, 1.5–5.5), and any hematologic effects (OR = 3.3; 95 % CI, (1.5–7.0). Almost three-quarters (68 %) of individuals residing in Ogale reported experiencing any current health symptoms compared to 56 % of Eteo residents. Chi-square analyses revealed that Ogale residents were significantly more likely to report having a headache (36 % vs. 18 %), dizziness (10 % vs. 2 %), throat irritation (8 % vs. 1 %), skin irritation (26 % vs. 5 %), rash (9 % vs. 1 %) and anemia (18 % vs. 4 %). After controlling for age, sex, and smoking, Ogale residents were significantly more likely to report having a headache (OR = 2.7; 95 % CI, 1.4–5.4), dizziness (OR = 6.3; 95 % CI, 1.3–30.4), eye irritation (OR = 2.5; 95 % CI, 1.1–5.7), throat irritation (OR = 9.1; 95 % CI, 1.1–75.4), skin irritation (OR = 6.5; 95 % CI, 2.4–18.2), and any type of anemia (OR = 5.9; 95 % CI, 1.9–18.3).

**Table 6:** Detailed multivariate analysis for self-reported health symptoms in Ogale and Eteo

Health symptom	Ogale (*n* = 100)	Eteo (*n* = 100)	Crude OR (95 % CI)	Adjusted OR^a^ (95 % CI)
Any symptom	68	56	1.6 (0.9, 3.0)	1.8 (1.0, 3.3)
Irritation*	47	25	2.7 (1.5, 4.8)	2.7 (1.5, 5.1)
Eye*	21	11	2.2 (0.9, 4.7)	2.5 (1.1, 5.7)
Throat*	8	1	8.6 (1.1, 70.2)	9.1 (1.1, 75.4)
Skin*	26	5	6.7 (2.5, 18.2)	6.6 (2.4, 18.2)
Rash	9	1	9.8 (1.2, 78.8)	NA
Runny nose	14	10	1.5 (0.6, 3.5)	1.5 (0.6, 3.5)
Cough	14	7	2.2 (0.8, 5.6)	2.5 (0.9, 6.5)
Gastrointestinal	17	9	2.1(0.9, 4.9)	2.0 (0.8,5.0)
Stomach pain	15	6	2.8 (1.0, 7.4)	2.7 (1.0, 7.2)
Diarrhea	2	4	0.5 (0.1, 2.8)	NA
Neurologic*	40	21	2.5 (1.3, 4.7)	2.8 (1.5, 5.5)
Headache*	36	18	2.6 (1.3, 4.9)	2.7 (1.4, 5.4)
Sleepiness	7	3	2.4 (0.6, 9.7)	NA
Dizziness*	10	2	5.4 (1.2, 25.5)	6.3 (1.3, 30.4)
Hematologic*	30	13	2.9 (1.4, 6.0)	3.3 (1.5, 7.0)
Anemia*	18	4	5.3 (1.7, 16.2)	5.9 (1.9, 18.3)
Malaria	14	9	1.7 (0.7, 4.0)	1.6 (0.7, 4.0)
General pain	8	11	0.7 (0.3, 1.8)	0.8 (0.3, 2.0)

*Abbreviations*: *OR* Odds Ratio, *NA* Not Applicable, odds ratio was not adjusted due to low event frequency in that category

^a^General health symptom categories, displayed in boldface, were adjusted for age, sex, smoking status, occupation, and education level. Due to small sample sizes, specific symptoms were adjusted for age, sex and smoking status only

*Adjusted odds ratios are statistically significant at *P* < 0.05

## Discussion

This study is one of few to examine general population exposure to highly elevated concentrations of refined oil in drinking water. Prior research has focused mainly on crude oil exposures in occupational cohorts. Only one previous study [26] examined the relationship between petroleum contamination and adverse health outcomes in the Niger Delta region. Participants in Ogale were more likely to report symptoms indicative of central nervous system toxicity, including headaches and dizziness, consistent with the literature on occupational exposures to crude oil spills and on occupational exposures to VOCs [5, 6, 18]. Unlike the previous Ogoniland study and several studies of occupational exposures [17, 18, 25], Ogale residents did not report a greater prevalence of respiratory symptoms. Participants in Ogale were more likely to report throat irritation, skin irritation, and rashes; these symptoms are consistent with exposure to high concentrations of PAHs and VOCs found in oil [5, 6, 12]. In addition, a significantly higher proportion of participants in Ogale reported a diagnosis of anemia, as might be expected from exposures to benzene and naphthalene [9, 10, 12, 16], although residents did not report the specific type of anemia with which they were diagnosed. More than 80 % of Ogale participants report that they still use untreated water on a daily basis.

Responses to the contamination in Ogale have focused on the delivery of clean drinking water supplies, rather than on remediation of the water supply. However, these efforts have not proved to be efficacious: the frequency of emergency water delivery in the participants sampled ranged from daily to infrequently, suggesting instability in emergency water delivery. Only one quarter of the participants in Ogale reported receiving emergency water and, of these, only half found the emergency drinking water supply to be sufficient for their daily needs. On average, only 15 % reported using emergency water as a primary source for their daily household activities. There were no significant differences in self-reported symptoms between Ogale participants who reported their primary drinking water source as borehole water, sachet water or emergency water. This result might be due to a number of factors, such as: (1) Ogale participants who receive inadequate emergency water supplies may still be exposed to contaminated borehole water. Even if the emergency supply is adequate for drinking and cooking, residents of Ogale might be exposed to petroleum hydrocarbons via inhalation and dermal routes. Inhalation and dermal exposures may occur through use of contaminated water for household activities such as bathing and cleaning; (2) Emergency water may not have been in use long enough to alleviate symptoms that might be associated with drinking borehole water; (3) Sachet water might also be contaminated. Prior studies on sachet water quality in Nigeria have found numerous chemical and bacterial contaminants, as well as widespread improper storage and handling practices [31]; or (4) It is also possible that not all borehole water in Ogale is contaminated; UNEP did not precisely define the limits of contamination.

The present study is limited by a relatively small sample size; however, participants in both communities were selected via a random sampling technique to increase study generalizability. In addition, our high response rates – 98 % in both communities – indicate that sampling bias is unlikely. Because our adjustment for confounders was limited by our small sample size, the possibility of residual confounding remains. Our cross-sectional design makes it difficult to infer causation for the association between petroleum contamination and adverse health effects.

Our results may be a consequence of our small sample size. They may also have been affected by misclassification of exposures and outcomes. State-of-the-science biomarker evaluation of exposures to petroleum was not feasible for this pilot work due to infrastructure and security constraints. Participants who reported use of emergency water may also be drinking borehole water at work or school. We were unable to measure petroleum hydrocarbon concentrations directly in the households surveyed; instead, our dichotomous classification of exposure was based upon UNEP’s analytical data indicating the location of contaminated drinking water. It is also possible that Ogale and Eteo differ with respect to non-borehole water sources of petroleum hydrocarbons, although no such differences are evident.

Self-report bias is a limitation of our outcome classification, as participants in Ogale were aware of the water supply contamination. This may have resulted in an overestimation of the observed association. We attempted to minimize self-report bias by masking the study objectives and hypothesis from participants. To avoid prompting, participants who reported experiencing any current health symptoms were asked to describe the health symptoms to the interviewers. This method was used to decrease the probability of information bias.

## Conclusions

In this cross-sectional pilot study, the first carried out in response to the UNEP recommendations, we observed statistically significant associations between exposure to petroleum-contaminated drinking water and self-reported symptoms consistent with exposure to petroleum hydrocarbons. These results reinforce UNEP’s recommendations for establishment of a health registry, medical surveillance, and a prospective cohort study for the Ogale community [3]. Future studies should define the full extent of contaminated household water and incorporate more detailed methods of exposure and outcome assessment for exposed populations, including its most susceptible members.
